# Ru-Catalyzed Reverse Water Gas Shift Reaction with Near-Unity Selectivity and Superior Stability

**DOI:** 10.1021/acsmaterialslett.1c00523

**Published:** 2021-10-27

**Authors:** Rui Tang, Zhijie Zhu, Chaoran Li, Mengqi Xiao, Zhiyi Wu, Dake Zhang, Chengcheng Zhang, Yi Xiao, Mingyu Chu, Alexander Genest, Günther Rupprechter, Liang Zhang, Xiaohong Zhang, Le He

**Affiliations:** † Institute of Functional Nano & Soft Materials (FUNSOM), Jiangsu Key Laboratory for Carbon-Based Functional Materials & Devices, Joint International Research Laboratory of Carbon-Based Functional Materials and Devices, Soochow University, Suzhou, Jiangsu 215123, China; ‡ Institute of Materials Chemistry, Technische Universität, Wien, Vienna 1060, Austria

## Abstract

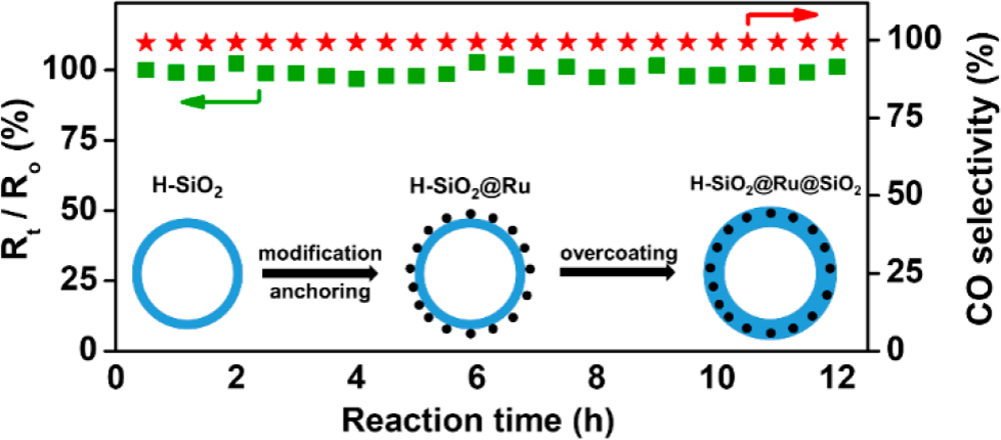

Cascade catalysis of reverse water gas shift (RWGS) and well-established CO hydrogenation holds promise for the conversion of greenhouse gas CO_2_ and renewable H_2_ into liquid hydrocarbons and methanol under mild conditions. However, it remains a big challenge to develop low-temperature RWGS catalysts with high activity, selectivity, and stability. Here, we report the design of an efficient RWGS catalyst by encapsulating ruthenium clusters with the size of 1 nm inside hollow silica shells. The spatially confined structure prevents the sintering of Ru clusters while the permeable silica layer allows the diffusion of gaseous reactants and products. This catalyst with reduced particle sizes not only inherits the excellent activity of Ru in CO_2_ hydrogenation reactions but also exhibits nearly 100% CO selectivity and superior stability at 200–500 °C. The ability to selectively produce CO from CO_2_ at relatively low temperatures paves the way for the production of value-added fuels from CO_2_ and renewable H_2_.

The interest in heterogeneous catalytic hydrogenation of CO_2_ is driven by the urgency to reduce our reliance on fossil fuels.^1−9^ This process utilizes CO_2_ from burning fossil fuels and renewable H_2_ to produce fuels and feedstock chemicals toward a carbon-neutral economy. It also provides a promising solution to the cost and safety issues associated with the storage, transportation, and distribution of H_2_ to facilitate the development of green H_2_ economy.^10−13^ Among different CO_2_ hydrogenation processes, the conversion of CO_2_ to CO via the reverse water gas shift (RWGS) reaction (CO_2_ + H_2_ ⇋ CO + H_2_O) is attracting more and more attention.^14−16^ As a highly valuable component of syngas, CO could be used to synthesize liquid hydrocarbons through the Fischer–Tropsch synthesis.^17,18^ Compared with direct CO_2_ hydrogenation, cascade catalysis of RWGS and CO hydrogenation could increase the yield of methanol.^19,20^ As outline in the liquid sunshine roadmap, methanol is one of optimal energy reservoirs for storing hydrogen and electricity. Moreover, CO is also a key feedstock for many organic chemicals such as acids, esters, and amines.^21−24^

To date, various RWGS catalysts have been developed, such as noble and transition metals, as well as metal oxides, carbides, and phosphides.^25−31^ Most existing catalysts produce CO with a high yield and selectivity only at temperatures above 500 °C.^32−34^ At high temperatures, the endothermic RWGS process is thermodynamically more favorable than the competing exothermic Sabatier reaction (CO_2_ + 4H_2_ ⇋ CH_4_ + 2H_2_O). When the methanation reaction follows the RWGS pathway, the increase of reaction temperature accelerates the desorption of CO intermediate and thus kinetically inhibits its further hydrogenation to produce methane. Nevertheless, the high-temperature RWGS reaction translates to a high-energy consumption and rapid catalyst deactivation caused by sintering.^35−37^ On the other hand, cascade catalysis of low-temperature RWGS and well-established CO hydrogenation holds promise for the conversion of greenhouse gas CO_2_ into liquid hydrocarbons and methanol under mild conditions. It is thus highly desired to achieve an effective CO production via the low-temperature RWGS reaction with a high rate and selectivity.^38^

Intuitively, catalysts with strong hydrogenation ability are preferred to improve the activity in low-temperature RWGS reactions. In practice, they could exhibit a very poor selectivity to CO production at low temperatures. For example, ruthenium is renowned as one of the most active catalysts for CO_2_ hydrogenation reactions but often favors the production of CH_4_.^39,40^ Recent studies suggest that the selectivity of Ru-based RWGS catalysts is strongly dependent on the metal dispersity.^41−45^ Supported Ru clusters with the size of <2 nm could selectively convert CO_2_ to CO while Ru nanoparticles with larger sizes produce CH_4_. DFT modeling showed that bare Ru (0001) surfaces facilitate the direct production of CO through C–O bond cleavage, which requires constant removal of surface oxygen to keep this pathway active.^46,47^ Because of their high surface energy and low Taman temperatures, Ru clusters are, however, vulnerable to rapid deactivation through sintering.^42,43^ Zeng and co-authors reported that small-sized Ru nanoparticles (1–3 nm) encapsulated within mesoporous silica (m-SiO_2_) nanowires catalyzed the RWGS reaction with CO selectivity of ∼100% at 350 °C and 93.4% at 400 °C. It was also demonstrated that the Ru@m-SiO_2_ catalyst with Ru loading of 1.6% could maintain long-term CO selectivity of 80% and 66% at 400 and 500 °C, respectively.^42^ Despite this progress, it is highly desired but challenging to develop Ru-based low-temperature RWGS catalysts that could exhibit a complete selectivity to CO formation and long-term durability in a wide temperature range.^48^

Here, we report the design of an efficient RWGS catalyst by encapsulating Ru clusters with the size of 1 nm inside hollow silica shells. The spatially confined structure prevents the sintering of Ru clusters, which is responsible for the high catalyst stability at 400 °C. H_2_ and CO_2_ gain access to the encapsulated Ru clusters via diffusion through the silica shell to produce CO and H_2_O, which leave the hollow catalyst through the silica layer.^49^ This catalyst with reduced particle sizes not only maintains the superior activity of Ru in CO_2_ hydrogenation reactions but also exhibits nearly 100% CO selectivity at 200–400 °C by inhibiting the methanation reaction.

The key of our strategy is to stabilize Ru nanoclusters by encapsulating them inside a hollow sol–gel silica shell. Figure S1 in the Supporting Information illustrates the preparation process of sandwich-like H-SiO_2_@Ru@SiO_2_ structures, which includes the deposition of Ru nanoclusters onto the surface of hollow silica spheres and subsequent coating by an outer silica layer. In a previous study, ultrafine Ru nanoparticles (1–3 nm) were confined within the mesoporous of silica nanowires.^42^ Nevertheless, the Ru size was smaller than the average pore size (4.1 nm) and particle sintering could still occur, which was responsible for unsatisfactory stability and CO selectivity at temperatures above 350 °C. In contrast, the solid silica shell without mesopores provides an improved spatially confined environment to protect Ru nanoclusters against sintering. It is also important to note that the thin and hollow sol–gel silica shell is permeable to gaseous reactants (CO_2_ and H_2_) and products (CO and H_2_O), which is apparently necessary for the catalytic RWGS reaction to occur on Ru surfaces.

Experimentally, a green salt-templated method was first used to synthesize hollow silica spheres with an average inner diameter of 474 nm and thickness of 20 nm (Figures 1a and 1b). Hollow silica spheres were chosen as the supports, because of their higher specific surface areas and better mass transfer than solid ones.^50,51^ The hollow silica was further functionalized with amine groups before the adsorption of Ru nanoclusters with an average size of 1 nm (Figure S2 in the Supporting Information). Figures 1c and 1d depicts transmission electron microscopy (TEM) images of Ru decorated hollow silica spheres, denoted as H-SiO_2_@Ru. The H-SiO_2_@Ru particles were well-dispersed without any aggregation. Because of their small size, Ru clusters can barely be observed in the low-magnification image of H-SiO_2_@Ru (Figure 1c). The enlarged TEM image nevertheless clearly shows the successful loading of well-dispersed ultrafine Ru nanoparticles (Figure 1d), which is further confirmed by elemental mapping (see Figure S3 in the Supporting Information). Because of the low Ru loading (∼1%) and very small particle size, no characteristic peaks of metallic Ru were found in the X-ray diffraction (XRD) patterns of H-SiO_2_@Ru, which is consistent with our previous study (Figure S4 in the Supporting Information).^52^

**Figure 1:**
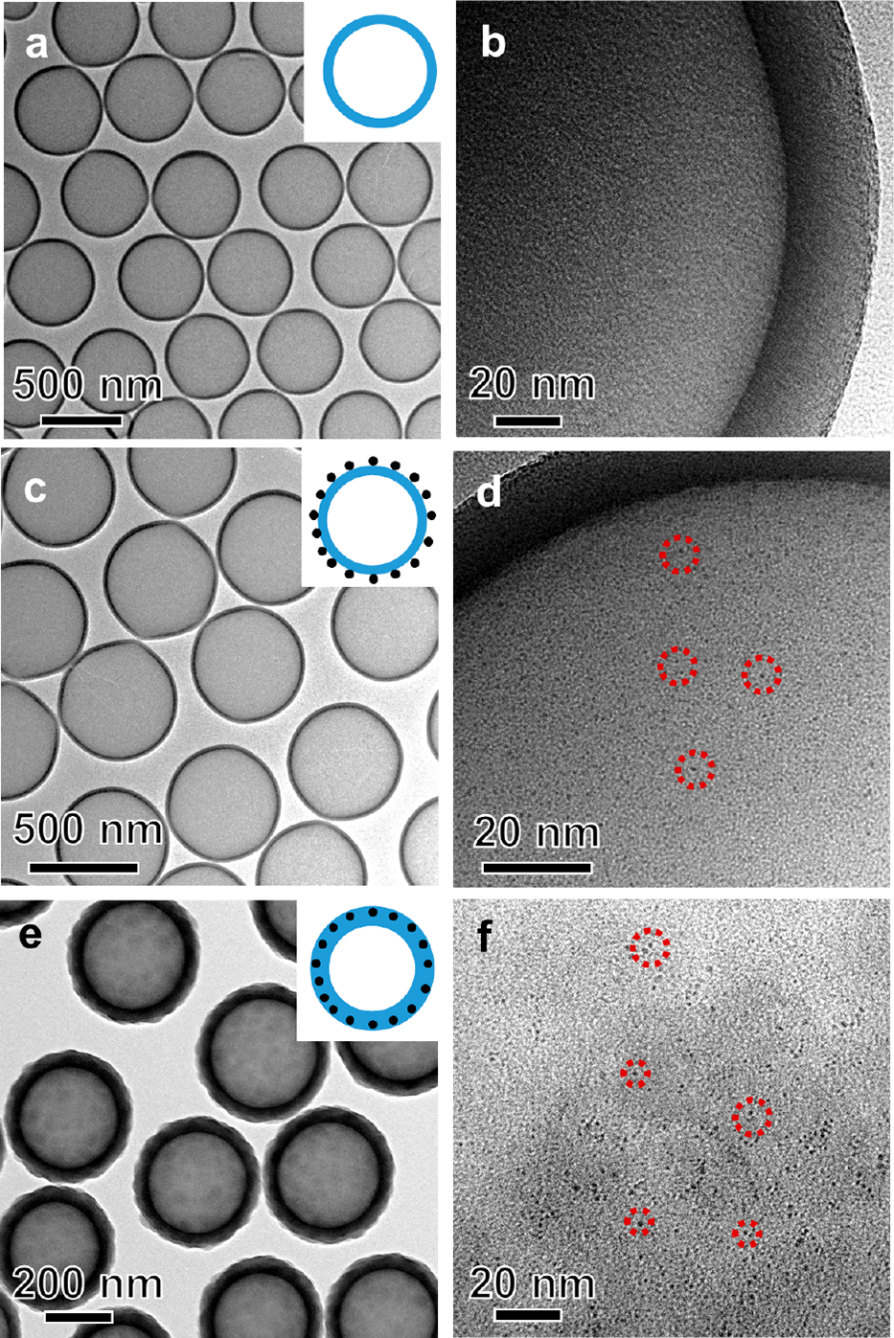
TEM images of prepared hollow nanoparticles: (a, b) H-SiO_2_, (c, d) H-SiO_2_@Ru, and (e, f) H-SiO_2_@Ru@SiO_2_-30.

Next, the as-obtained H-SiO_2_@Ru particles were overcoated with an outer layer of silica through the sol–gel method to form a sandwich-like structure. Figures 1e and 1f shows TEM images of the as-obtained sample, denoted as H-SiO_2_@Ru@SiO_2_-30, after deposition of a 30-nm-thick silica coating on H-SiO_2_@Ru. The H-SiO_2_@Ru@SiO_2_-30 particles remained monodisperse while the shell thickness increased uniformly, suggesting the successful coating on individual H-SiO_2_@Ru particles. High-resolution TEM images confirmed the presence of well-dispersed Ru nanoclusters with the original size (see Figure 1f, as well as Figure S5 in the Supporting Information). Moreover, elemental mapping results confirmed that Ru clusters were sandwiched between two silica layers (Figure S6 in the Supporting Information). After the outer silica coating, the Ru loading determined by inductively coupled plasma–mass spectrometry (ICP-MS) decreased to 0.48 wt % for H-SiO_2_@Ru-30. These results clearly reveal the successful preparation of sandwich-like H-SiO_2_@Ru@SiO_2_ nanostructures.

To investigate the effect of the outer silica shell on the stability of Ru nanoclusters against sintering, H-SiO_2_@Ru and H-SiO_2_@Ru@SiO_2_-30 samples were pretreated at 500 °C in H_2_ for 4 h. This high-temperature pretreatment also removes the residual organic species in the catalysts. Figure 2a shows the TEM image of the H_2_-treated H-SiO_2_@Ru sample, denoted as H-SiO_2_@Ru–H_2_. The overall hollow morphology was well-preserved but the size of Ru increased sharply, as revealed by the observation of large Ru nanoparticles in the size range of 4–6 nm. Figure 2b depicts the high-resolution TEM image of a single Ru nanoparticle. The lattice spacing along a specific direction was found to be 0.205 nm, which is in agreement with the (101) crystal plane of hexagonal Ru (JCPDS File No. 89-4903). The average size of Ru particles increased from 1 nm to 2.3 nm after the pretreatment (Figure S7 in the Supporting Information). In contrast, no obvious sintering of Ru clusters occurred in the presence of the outer silica shell (Figures 2c and 2d). The average size of Ru in the treated sandwiched structures, denoted as H-SiO_2_@Ru@SiO_2_-30–H_2_, was the same as that of H-SiO_2_@Ru@SiO_2_-30 (Figure S8 in the Supporting Information). These results clearly reveal that the outer silica shell of the sandwiched structure plays a critical role in stabilizing Ru clusters against sintering.

**Figure 2:**
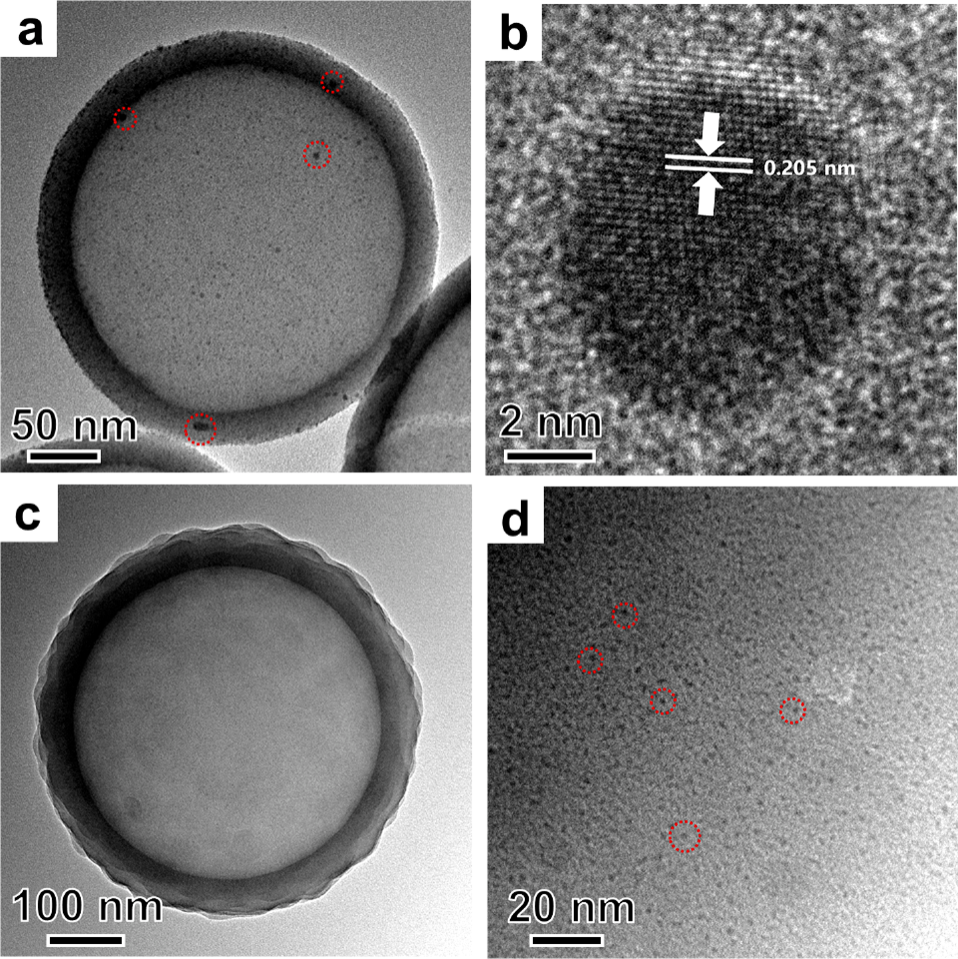
TEM images of different catalysts after H_2_ treatment at 500 °C: (a, b) H-SiO_2_@Ru–H_2_ and (c, d) H-SiO_2_@Ru@SiO_2_-30–H_2_.

As shown above, the sandwiched structure design greatly improves the sintering resistance of Ru clusters, which is the key to achieve high activity, selectivity, and stability in catalyzing the RWGS reaction. The performance of H-SiO_2_@Ru–H_2_ and H-SiO_2_@Ru@SiO_2_-30–H_2_ in catalyzing low-temperature RWGS reactions was investigated in a flow reactor at ambient pressure. The feed ratio of CO_2_:H_2_:N_2_ was kept at 1:1:2, and the reaction temperatures were varied from 200 °C to 400 °C. It is important to note that the degree of CO_2_ conversion was kept much lower than the equilibrium conversion at the specific temperature in all experiments (see Figure S9 and Table S2 in the Supporting Information). The CO_2_ conversion rate of H-SiO_2_@Ru–H_2_, *R*_CO_2__ increased as the temperature increased, but the CO selectivity only reached ∼80% with CH_4_ as the only byproduct within the investigated temperature range (Figures 3a and 3b). The formation of CH_4_ is ascribed to the presence of relatively large-sized Ru nanoparticles that favor complete hydrogenation of CO_2_.^42,45^ In distinct contrast, the cluster catalyst of H-SiO_2_@Ru@SiO_2_–H_2_ exhibited a nearly 100% CO selectivity throughout the temperature range of 200–400 °C (see Figures 3c and 3d). Further hydrogenation of CO was inhibited for Ru clusters with the size of ∼1 nm, which is consistent with previous studies.^41−45^*R*_CO_2__ of H-SiO_2_@Ru@SiO_2_-30–H_2_ was slightly lower than that of H-SiO_2_@Ru–H_2_, which might be attributed to the extra diffusion of reactants and products through the outer silica shell. It is important to note that H-SiO_2_@Ru@SiO_2_-30–H_2_ outperformed reported low-temperature Ru catalysts, in terms of both the activity and selectivity (see Table S1 in the Supporting Information).

**Figure 3:**
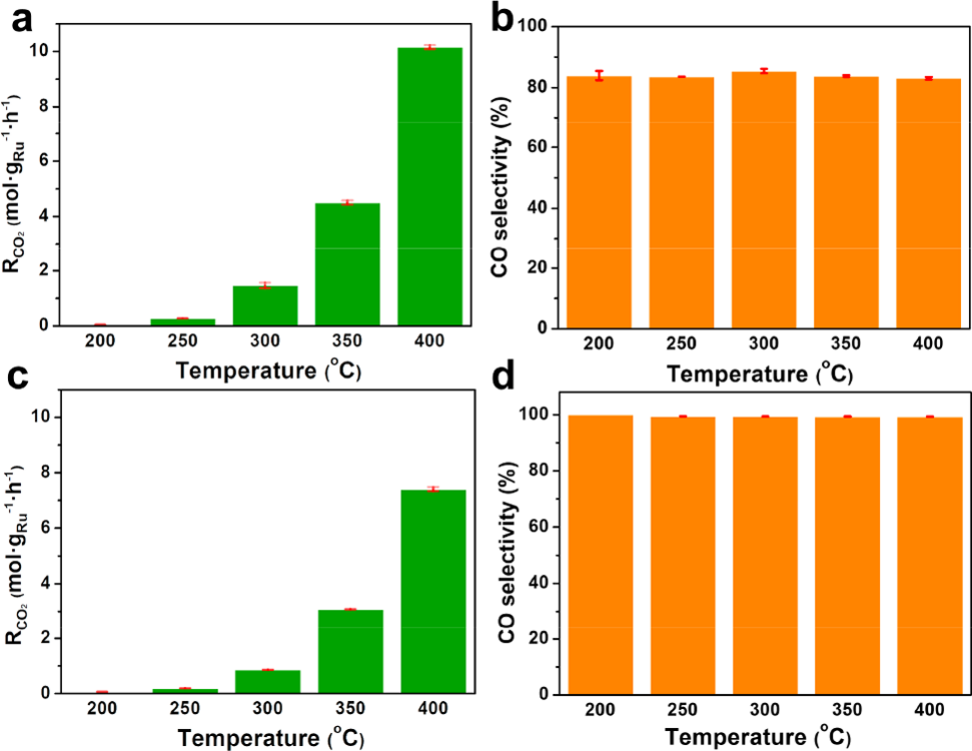
Temperature-dependent activity and selectivity of (a, b) H-SiO_2_@Ru–H_2_ and (c, d) H-SiO_2_@Ru@SiO_2_-30–H_2_ in catalyzing CO_2_ hydrogenation.

While Ru clusters have been demonstrated to exhibit high CO selectivity in catalyzing the RWGS reaction, it is often difficult to maintain a 100% CO selectivity during the long-term operation, because of the catalyst sintering. The spatial confinement in the sandwiched structure provides an effective way of stabilizing Ru clusters under reaction conditions. To demonstrate the superior catalyst stability of H-SiO_2_@Ru@SiO_2_-30–H_2_, the catalytic performance of both H-SiO_2_@Ru–H_2_ and H-SiO_2_@Ru@SiO_2_-30–H_2_ samples was tested in a continuous run of 12 h at 400 °C. In the absence of the outer silica shell, *R*_CO_2__ declined by 10.1% within 12 h while the CO selectivity remained below 85% for H-SiO_2_@Ru–H_2_ (Figures 4a and 4b). The slight increase of CO selectivity for H-SiO_2_@Ru–H_2_ during the stability test may be attributed to the decrease in the degree of CO_2_ conversion with time. In contrast, H-SiO_2_@Ru@SiO_2_-0.3–H_2_ exhibited very stable activity and nearly 100% CO selectivity during the 12-h period (see Figures 4c and 4d). Even by prolonging the reaction time to 30 h, the activity of H-SiO_2_@Ru@SiO_2_-30–H_2_ only decreased by 3.3% without changing the CO selectivity and the size of Ru particles remained below 2 nm (see Figures S10–14 in the Supporting Information).

**Figure 4:**
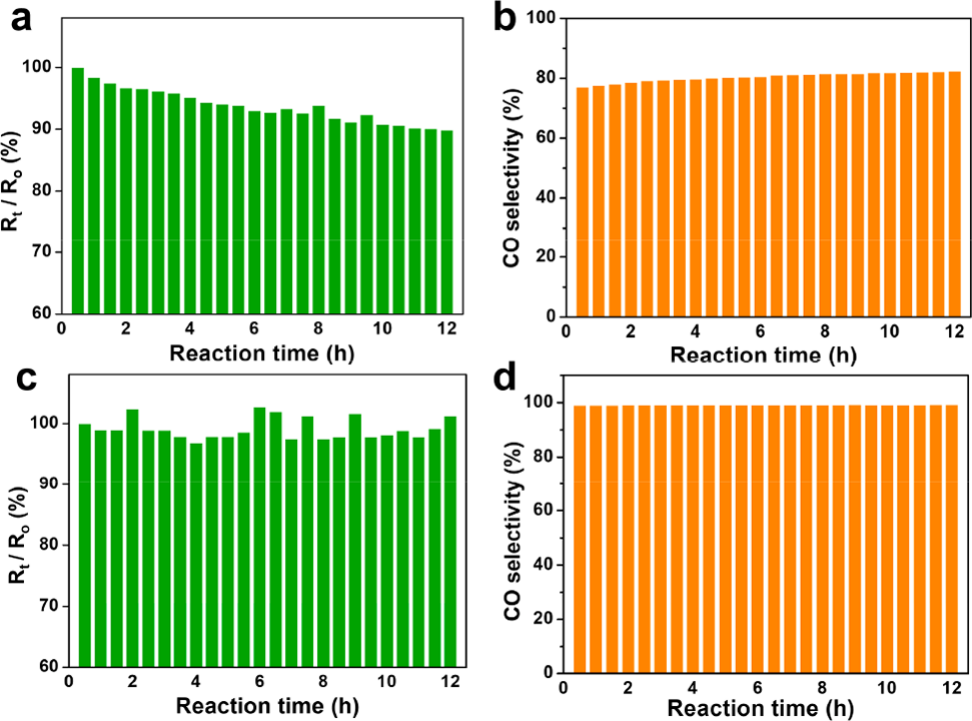
Catalytic stability of (a, b) H-SiO_2_@Ru–H_2_ and (c, d) H-SiO_2_@Ru@SiO_2_-30–H_2_ in a continuous 12-h run at 400 °C. *R*_0_ refers to *R*_CO_2__ at the beginning of the reaction. *R*_t_ refers to *R*_CO_2__ at different reaction times.

To understand the difference in catalyst stability, the spent catalysts of H-SiO_2_@Ru–H_2_ and H-SiO_2_@Ru@SiO_2_-30–H_2_ after the 12-h testing at 400 °C were investigated by TEM. Some large Ru particles in the size range of 6–12 nm were clearly observed in the spent sample of H-SiO_2_@Ru–H_2_. (See Figures 5a and 5b, as well as Figure S15 in the Supporting Information.) In contrast, no Ru particles in the similar size range were observed in the spent H-SiO_2_@Ru@SiO_2_-30–H_2_ catalyst (see Figures 5c and 5d, as well as Figure S16 and Table S3 in the Supporting Information). Moreover, H_2_ temperature-programmed desorption (H_2_-TPD) was also used to quantitively compare the Ru dispersity and, thus, the degree of sintering (see Figures S17 and S18 in the Supporting Information). All of the desorption peaks centered at 60–130 °C were attributed to the hydrogen adsorbed on the Ru species.^39,42,53^ The desorption peak area decreased sharply for H-SiO_2_@Ru–H_2_ before and after the catalysis, suggesting the loss of some active sites. The Ru dispersion decreased from 58% to 17% after the stability test, further confirming the obvious sintering in the absence of the outer silica shell. In contrast, the H_2_-TPD profiles were quite similar for fresh and spent H-SiO_2_@Ru@SiO_2_-30–H_2_ catalysts and the Ru dispersity only declined from 79% to 75%. These results clearly reveal the effectiveness of our strategy in stabilizing Ru clusters against sintering under catalytic conditions.

**Figure 5:**
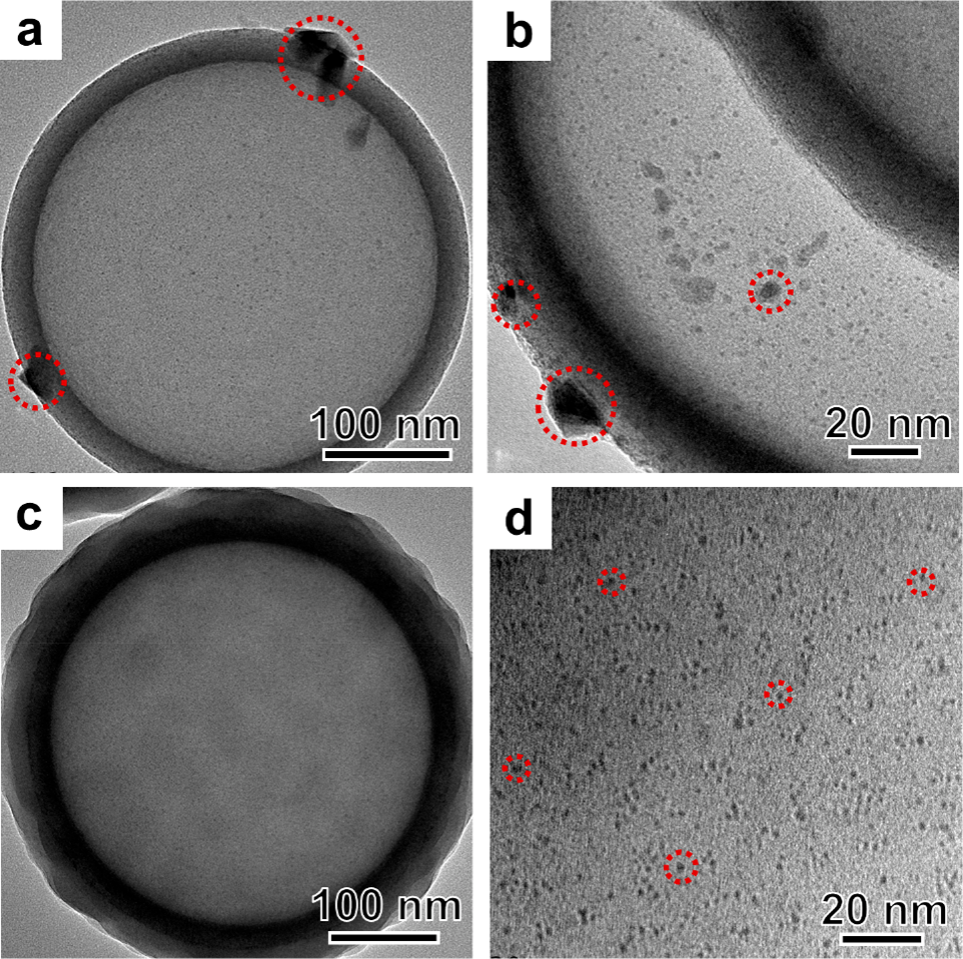
TEM images of catalysts after tested at 400 °C for 12 h: (a, b) H-SiO_2_@Ru–H_2_ and (c, d) H-SiO_2_@Ru@SiO_2_-30–H_2_.

As shown above, the encapsulation in the silica shell greatly enhances the stability of Ru clusters. The permeability of silica shells to gaseous products and reactants might be a major concern. While sol–gel silica is quite permeable, because of incomplete hydrolysis and condensation, the treatment at high temperature would increase the condensation degree and thus reduce its gas permeability. Nevertheless, the catalytic performance of H-SiO_2_@Ru@SiO_2_-30–H_2_ suggests that CO_2_ and H_2_ are able to diffuse through the 30-nm-thick outer silica shell pretreated at 500 °C and gain access to the Ru clusters.

To further demonstrate the permeability of the silica shell, we prepared another sandwiched sample, denoted as H-SiO_2_@Ru@SiO_2_-50, by increasing the thickness of the outer silica to 50 nm. Similar to H-SiO_2_@Ru@SiO_2_-30, Ru clusters were well-dispersed and maintained the original size after the silica coating (see Figures S19–S21 in the Supporting Information). Particle sintering was not observed in the sandwiched structure in the presence of the thicker silica shell after pretreatment in H_2_ at 500 °C (see Figures S22–S24 in the Supporting Information). The as-obtained sample, denoted as H-SiO_2_@Ru@SiO_2_-50–H_2_, exhibited stable activity and nearly 100% CO selectivity, which is similar to H-SiO_2_@Ru@SiO_2_-30–H_2_, in catalyzing the RWGS reaction (see Figures S25–S31 in the Supporting Information). In other words, the increase of shell thickness did not result in any loss of performance and showed better catalytic activity compared with H-SiO_2_@Ru@SiO_2_-30, which provides strong evidence in support of the required permeability of silica shell to small gas molecules. Apparently, the dispersity of H-SiO_2_@Ru@SiO_2_-50–H_2_ (85%) is higher than that of H-SiO_2_@Ru@SiO_2_-30–H_2_ (79%) (see Figure S32 and Table S3 in the Supporting Information). It is most likely that slight particle sintering still occurred for sandwiched structures. With the increase of the shell thickness, the stability of Ru particles against sintering is improved, responsible for the activity difference between the two catalysts.

The silica shell is permeable to gaseous reactants and products, even after treatment at higher temperatures. For example, the activity of both H-SiO_2_@Ru@SiO_2_-30–H_2_ and H-SiO_2_@Ru@SiO_2_-50–H_2_ catalysts further increased while the CO selectivity remained nearly 100% at the reaction temperature of 500 °C (see Figures S33 and S34 in the Supporting Information). Moreover, the H-SiO_2_@Ru@SiO_2_-50 catalyst pretreated at 800 °C in H_2_, denoted as H-SiO_2_@Ru@SiO_2_-50–800, maintained its original morphology and Ru dispersity (see Figures S35–S37 in the Supporting Information). Although such treatment reduced the permeability of the silica shell, H-SiO_2_@Ru@SiO_2_-50–800 still exhibited a high *R*_CO_2__ at 400 °C in catalyzing the RWGS reaction, which is 43.8% of that of the same catalyst pretreated at 500 °C (Figure S38 in the Supporting Information). More importantly, the CO selectivity of H-SiO_2_@Ru@SiO_2_-50–800 was still close to unity, because of the superior stability of Ru clusters against sintering (see Figure S39 and Table S2 in the Supporting Information).

In summary, we demonstrate an encapsulation strategy for the stabilization of 1 nm Ru clusters inside hollow silica shells. Notably, the silica shell without mesoscale pores is found to be highly permeable to gaseous reactants and products. This unique structure outperformed reported low-temperature Ru RWGS catalysts, in terms of activity, selectivity, and stability. Future work will focus on further optimization of the sandwich-structured catalyst, e.g., by increasing the Ru loading and permeability of the outer silica shell. It is believed that this universal encapsulation strategy could be easily extended to the preparation of sintering-resistant catalysts, based on other metal nanoparticles and clusters. The ability to efficiently and selectively produce CO from CO_2_ at relatively low temperatures paves the way for the development of cascade catalysis of RWGS and well-established CO hydrogenation reactions toward the production of value-added fuels and feedstock chemicals from CO_2_ and renewable H_2_ under mild conditions.

## Experimental Section

### Materials

All chemicals were used as received without further purification. Ruthenium chloride (RuCl_3_), sodium hydroxide (97% purity), and ethylene glycol (99.5% purity) were purchased from Aladdin. Sodium citrate (>98% purity), ammonium hydroxide (NH_3_**·**H_2_O, 28 wt %), tetraethyl orthosilicate (TEOS), 3-aminopropyltriethoxysilane (APTES, 99% purity), and ethanol were obtained from Energy Chemical (Shanghai), Macklin, Tokyo Chemical Industry, Acros and Sinopharm Chemical Reagent Co., Ltd., respectively. Milli-Q water (Millipore, 18.2 MΩ cm^–1^ at 25 °C) was used in all experiments.

### Preparation of Ru Nanoparticles

Ru nanoparticles were synthesized using a polyol-assisted method that was reported previously^54^ but with some modifications. In a typical synthesis, 0.10 g of RuCl_3_ and 0.20 g of sodium hydroxide were dissolved in 50 mL of ethylene glycol under continuous stirring. The solution was first heated at 80 °C for 30 min, and then at 160 °C for another 3 h. After cooling to room temperature, the as-obtained dispersion of Ru nanoparticles was collected for further use.

### Preparation of Hollow Silica Particles

Hollow SiO_2_ spheres with an inner diameter of 474 nm and a shell thickness of 20 nm were prepared through the salt-templated method.^55^ Briefly, 16 mL of H_2_O, 6.4 mL of NH_3_**·**H_2_O, and 8 mL of sodium citrate aqueous solution (0.2 M) were first added into 480 mL of ethanol under magnetic stirring. 0.7 mL of TEOS was then added into the solution. The reaction mixture was kept at room temperature for 12 h without stirring. The products were collected by centrifugation, cleaned two times with deionized water, and then dispersed in 5 mL of ethanol for further use.

### Synthesis of H-SiO_2_@Ru and H-SiO_2_@Ru@SiO_2_ Particles

Sandwich-like H-SiO_2_@Ru@SiO_2_ structures were prepared through the decoration of Ru nanoparticles onto the surface of hollow SiO_2_ particles and subsequent SiO_2_ coating. First, the as-obtained hollow SiO_2_ spheres (50 mg) were dispersed in a mixture of ethanol (40 mL) and APTES (1 mL). After stirring for 12 h, amine-functionalized H-SiO_2_ particles were collected by centrifugation, washed once with ethanol, and dispersed in 5 mL of ethanol. This dispersion was then mixed with 5 mL of the ethylene glycol solution containing Ru nanoclusters. The mixture was ultrasonicated for 15 min to allow the adsorption of Ru nanoclusters onto the surface of amine-functionalized hollow silica. The as-obtained H-SiO_2_@Ru particles were separated by centrifugation, washed once with ethanol, dispersed in a mixture of ethanol (80 mL), deionized (DI) water (12 mL), and NH_3_·H_2_O (4 mL). 0.3 mL of TEOS was slowly added into the reaction under magnetic stirring. After reacting for 3 h, the H-SiO_2_@Ru@SiO_2_-30 products were collected by centrifugation, washed with water and ethanol, and dried at 80 °C in an oven overnight. The H-SiO_2_@Ru@SiO_2_-50 sample was prepared via the same procedure, except for the use of 0.6 mL of TEOS.

### Characterization

Transmission electron microscopy (TEM) images were obtained using a TF20 FEI TEM system. The loadings of Ru in different samples were measured using an ICP-MS system (Aurora M90, Jenoptik). Powder X-ray diffraction (XRD) patterns were recorded on an Empyrean diffractometer with a Cu Kα radiation. Temperature-programmed desorption (TPD) was performed on an automatic chemical adsorption instrument (FINETEC/FINE-SORB-3010). For H_2_-TPD, ∼20 mg of sample was fixed in a U-shape quartz tube and flushed with Ar (40 mL/min) for 10 min, followed by heating to 300 °C (10 °C/min) for 60 min and then cooling to room temperature in the same Ar flow. Afterward, the sample was exposed to the adsorbate (e.g., H_2_) with a flow rate of 80 mL/min for 20 min at 25 °C and then flushed with Ar flow (40 mL/min) for 10 min. Finally, the sample was heated to 600 °C (10 °C/min) in the Ar flow (40 mL/min). The temperature and current for TCD were 60 °C and 90 mA, respectively.

### Catalytic Testing

Thermal catalytic CO_2_ hydrogenation experiments were performed in a quartz tube flow reactor with an inner diameter of 4 mm under atmospheric pressure. Fifteen milligrams (15 mg) of catalyst were loaded into the reactor tube and held in place by quartz wool for each test. The flow rates of feeding gases were fixed at 5 mL min^–1^ for CO_2_, 5 mL min^–1^ for H_2_, and 10 mL min^–1^ for N_2_. After separation by gas chromatography (Agilent, Model 7890B), the amounts of gas reactants and products were analyzed using a thermal conductivity detection (TCD) device and a flame ionization detection (FID) device (with a methanation unit).

## Supporting Information Available

Additional catalytic performance and characterization of the catalysts, including TEM, XRD, EDS elemental mapping, and H_2_-TPD (PDF)
