# Supplementary material for: Ru-Catalyzed Reverse Water Gas Shift Reaction with Near-Unity Selectivity and Superior Stability
Source: ACS Mater Lett. 2021 Oct 27;3(12):1652–9. doi: 10.1021/acsmaterialslett.1c00523 (PMC8653414; doi:10.1021/acsmaterialslett.1c00523)
Supplement: Supplementary file 1 — tz1c00523_si_001.pdf [file tz1c00523_si_001.pdf]

Ru-catalyzed reverse water gas shift reaction with near-unity selectivity and superior stability

Rui Tang,[1,†] Zhijie Zhu,[1,†] Chaoran Li,[1,] Mengqi Xiao,[1] Zhiyi Wu,[1] Dake Zhang,[1] Chengcheng Zhang,[1] Yi Xiao,[1] Mingyu Chu,[1] Alexander Genest,[2] Günther Rupprechter,[2] Liang Zhang,[1] Xiaohong Zhang,[1,*] Le He[1,*]*

[1]Institute of Functional Nano & Soft Materials (FUNSOM), Jiangsu Key Laboratory for Carbon-Based Functional Materials & Devices, Joint International Research Laboratory of Carbon-Based Functional Materials and Devices, Soochow University, Suzhou, Jiangsu 215123, China

[2]Institute of Materials Chemistry, Technische Universität Wien, Vienna 1060, Austria

[†]These authors contributed equally to this work

*Email: crli@suda.edu.cn; xiaohong_zhang@suda.edu.cn; lehe@suda.edu.cn

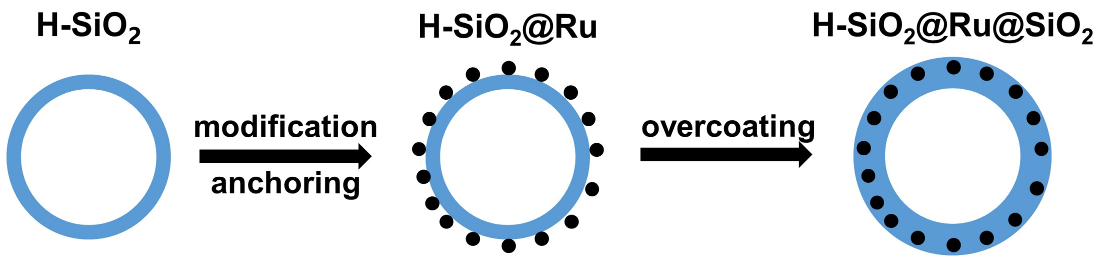

Figure S1. Schematic illustration of the synthesis process of H-SiO_2@Ru@SiO_2 nanostructures.

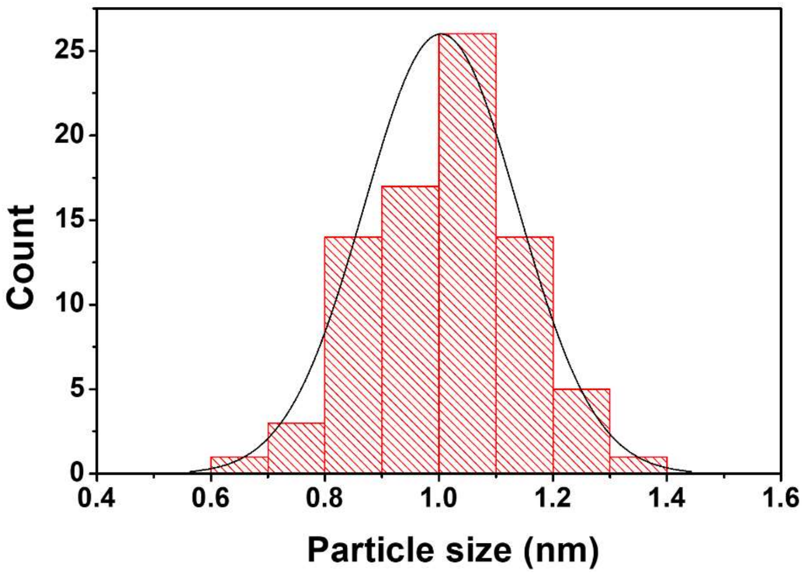

Figure S2. Size distribution of freshly prepared H-SiO_2@Ru.

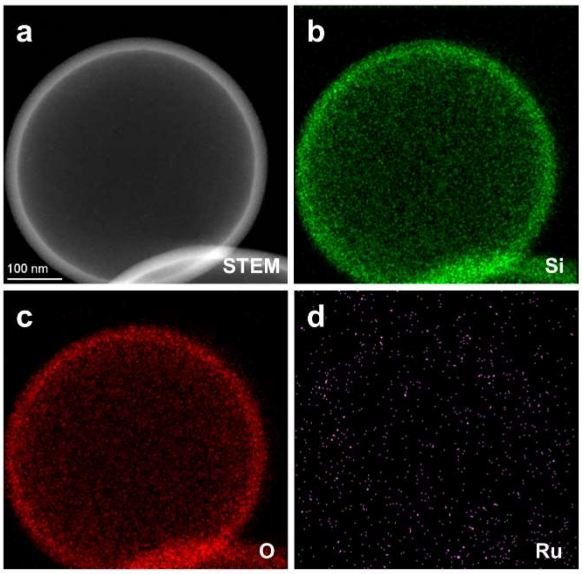

Figure S3. EDS elemental mapping images of H-SiO_2@Ru.

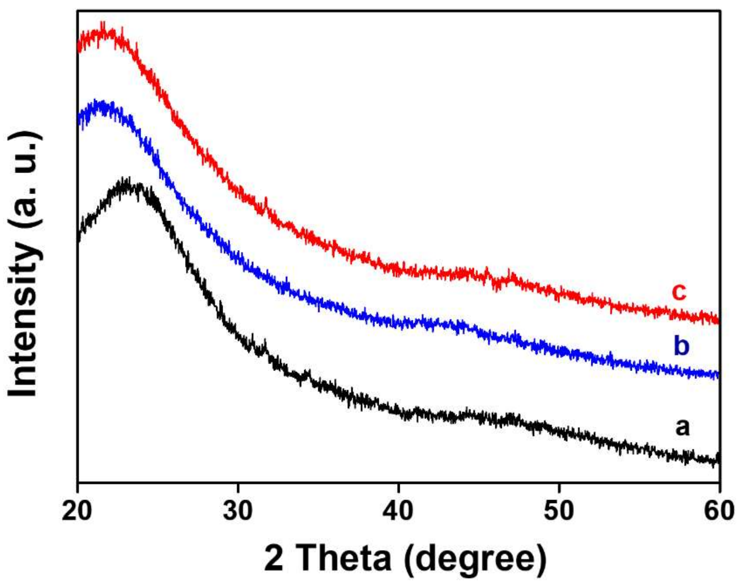

Figure S4. XRD patterns of H-SiO_2@Ru catalysts: (a) freshly prepared catalyst, (b) reduced catalyst and (c) spent catalyst.

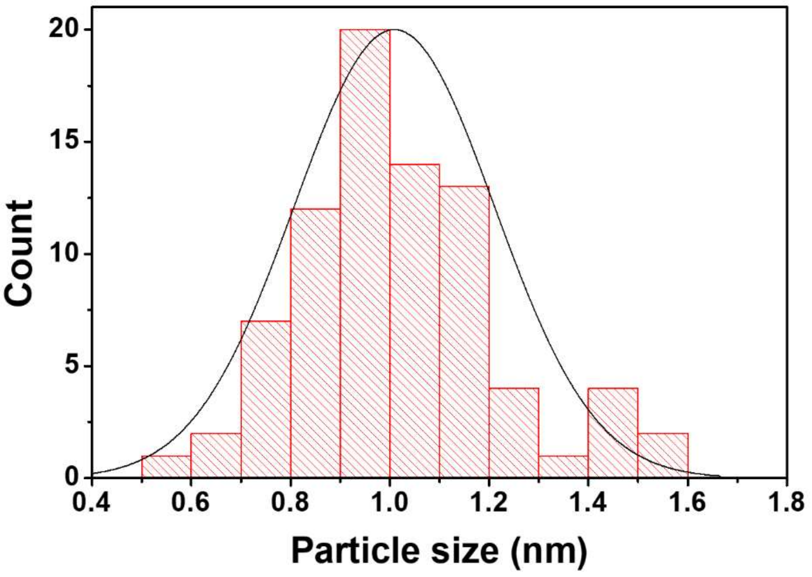

Figure S5. Size distribution of freshly prepared H-SiO_2@Ru@SiO_2-30.

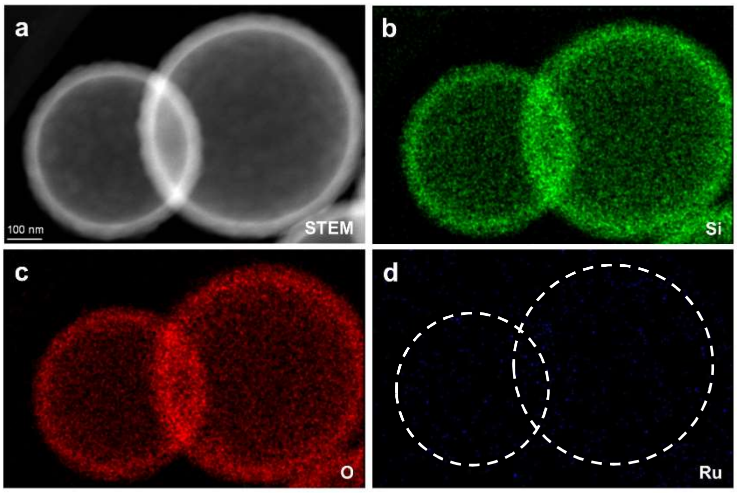

Figure S6. EDS elemental mapping images of H-SiO_2@Ru@SiO_2-30.

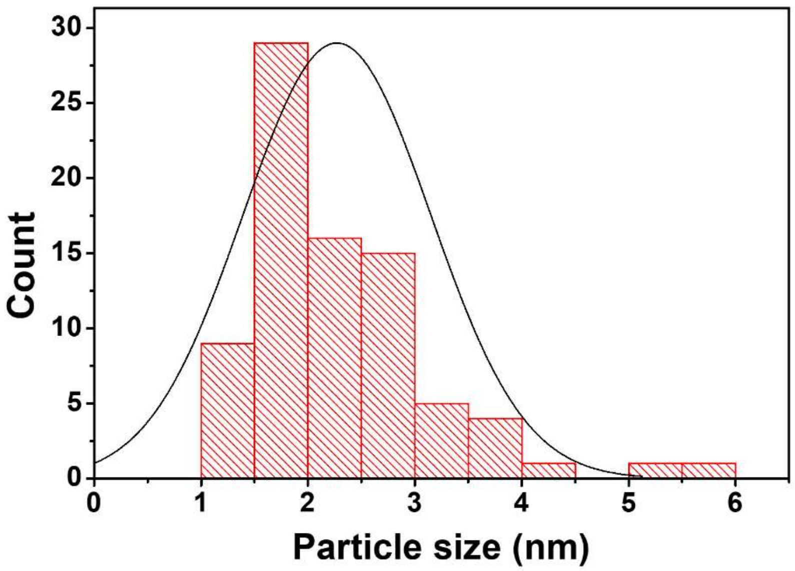

Figure S7. Size distribution of reduced H-SiO_2@Ru-H_2.

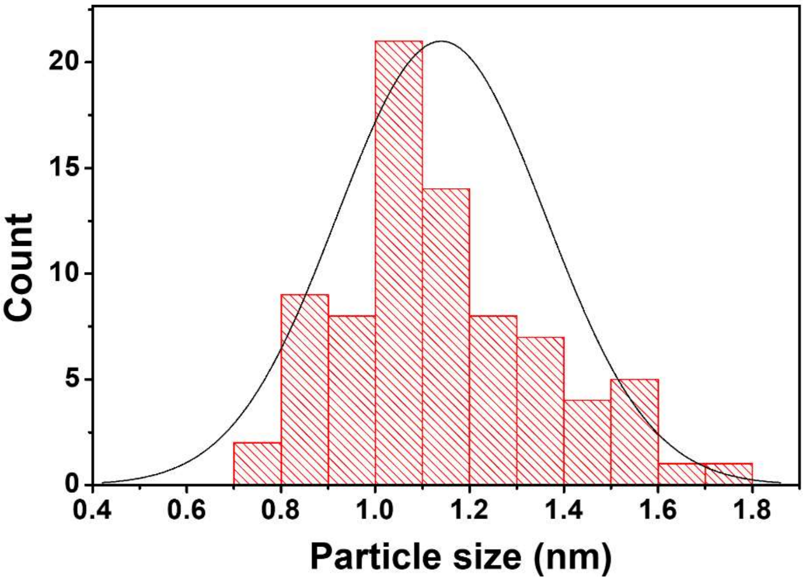

Figure S8. Size distribution of reduced H-SiO_2@Ru@SiO_2-30-H_2.

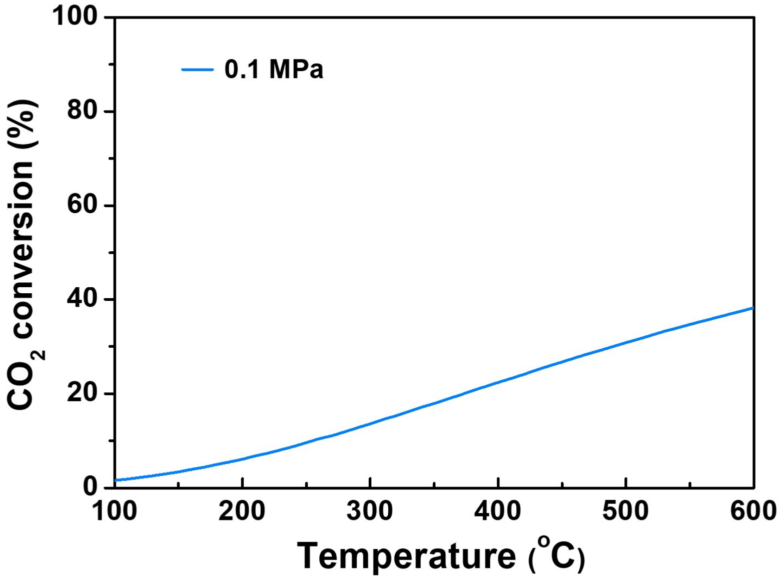

Figure S9. Thermodynamic CO_2 conversion degree at atmosphere pressure.

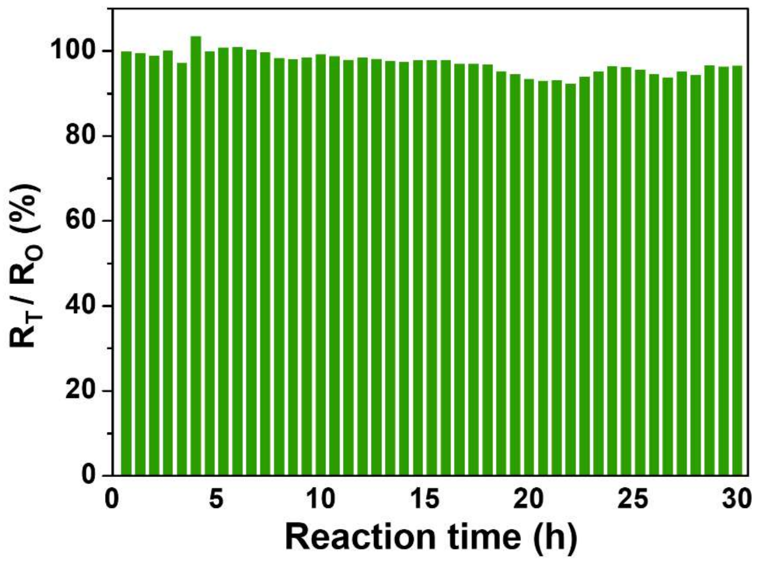

Figure S10. Catalytic activity of H-SiO_2@Ru@SiO_2-30-H_2 in a continuous 30-hour run at 400 °C.

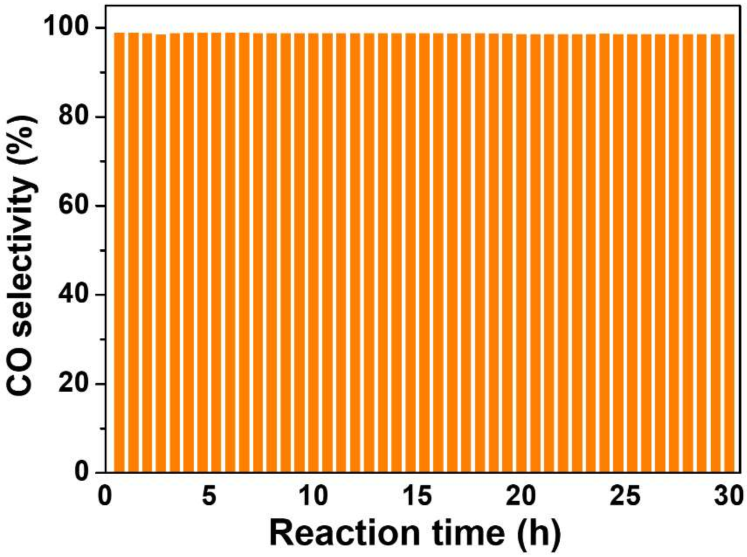

Figure S11. Catalytic selectivity of H-SiO_2@Ru@SiO_2-30-H_2 in a continuous 30-hour run at 400 °C.

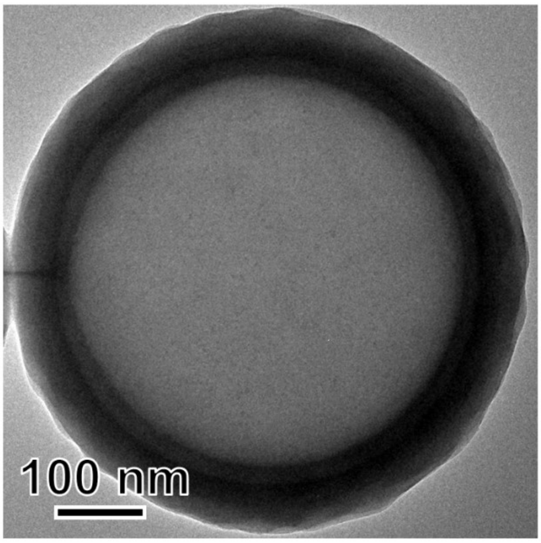

Figure S12. TEM image of H-SiO_2@Ru@SiO_2-30-H_2 after testing at 400 °C for 30 hours.

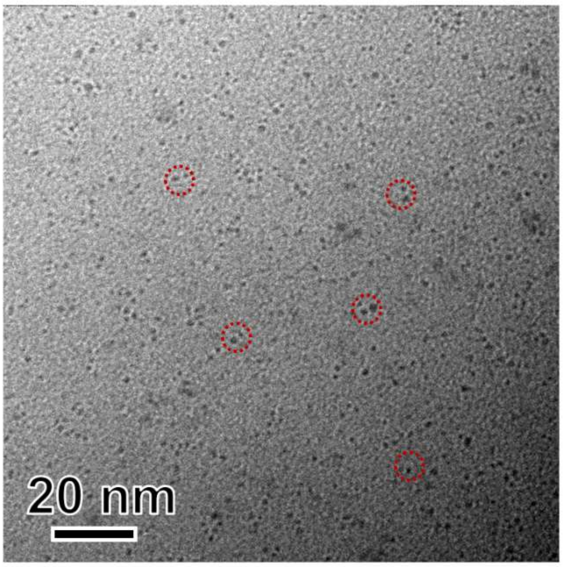

Figure S13. High-resolution TEM images of H-SiO_2@Ru@SiO_2-30-H_2 after testing at 400 °C for 30 hours.

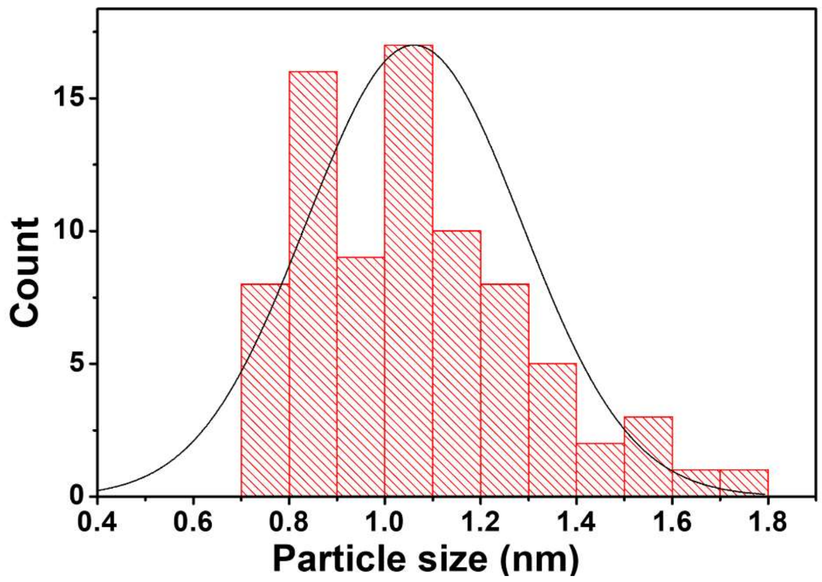

Figure S14. Size distribution of H-SiO_2@Ru@SiO_2-30-H_2 after testing at 400 °C for 30 hours.

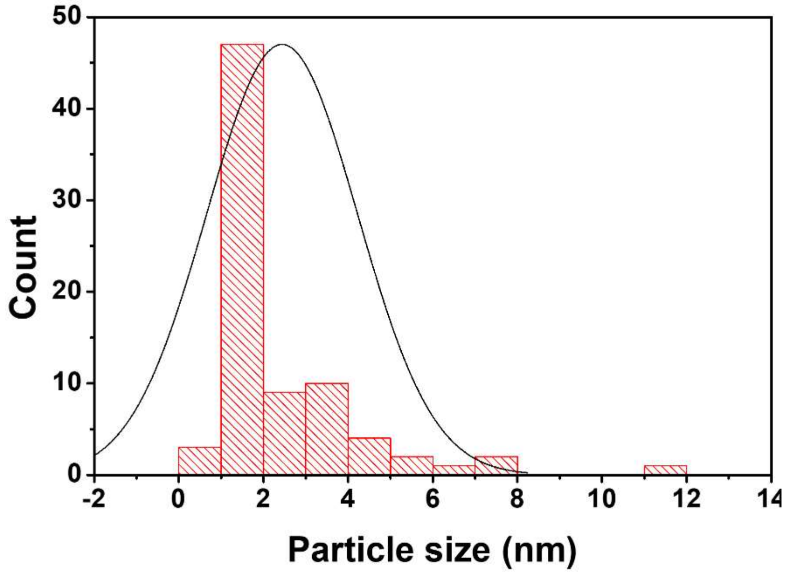

Figure S15. Size distribution of spent H-SiO_2@Ru-H_2.

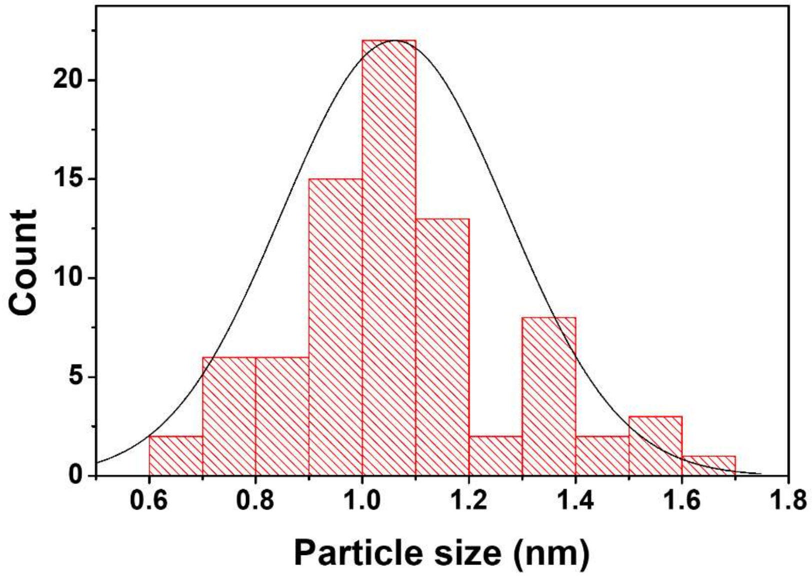

Figure S16. Size distribution of spent H-SiO_2@Ru@SiO_2-30-H_2.

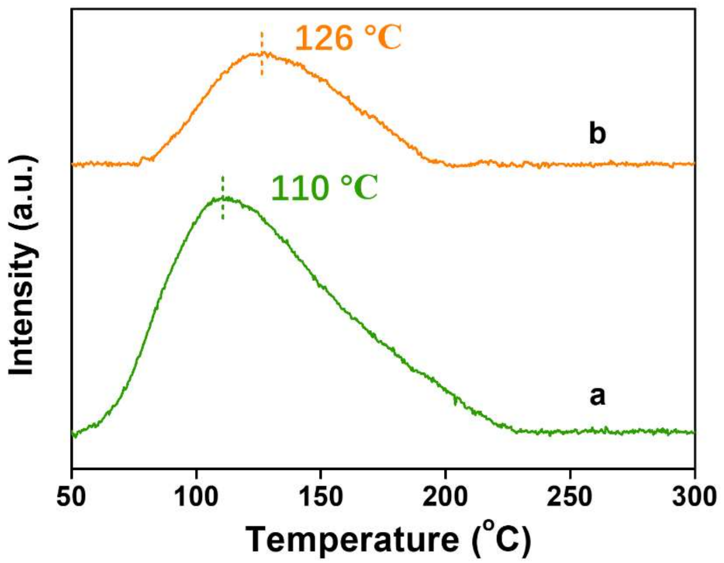

Figure S17. H_2-TPD profiles of (a) H-SiO_2@Ru-H_2, and (b) spent H-SiO_2@Ru-H_2.

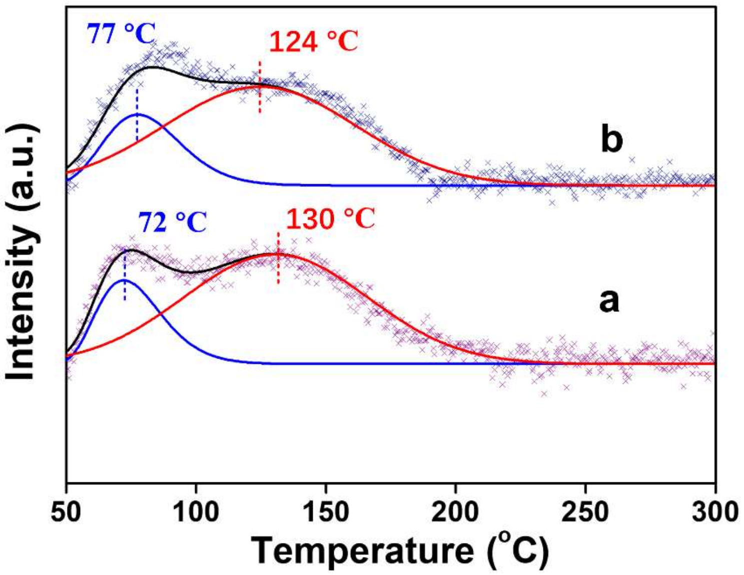

Figure S18. Hydrogen temperature programmed desorption (H_2-TPD) profile: (a) H-SiO_2@Ru@SiO_2-30-H_2, (b) spent H-SiO_2@Ru@SiO_2-30-H_2.

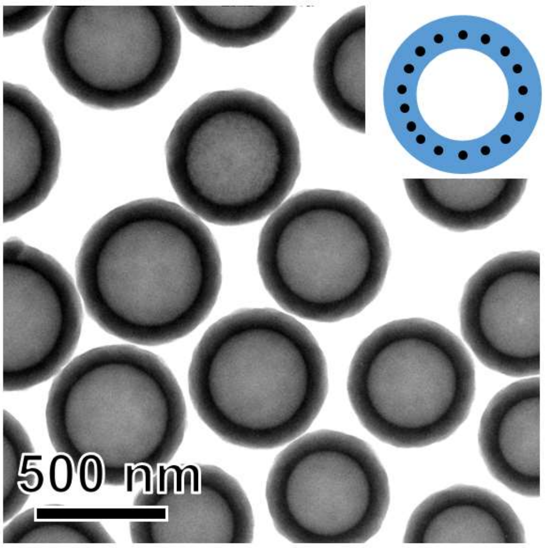

Figure S19. TEM images of freshly prepared H-SiO_2@Ru@SiO_2-50.

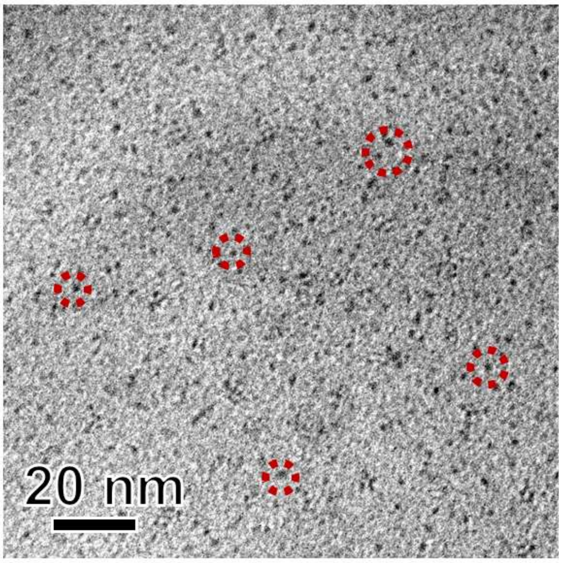

Figure S20. High-resolution TEM image of freshly prepared H-SiO_2@Ru@SiO_2-50.

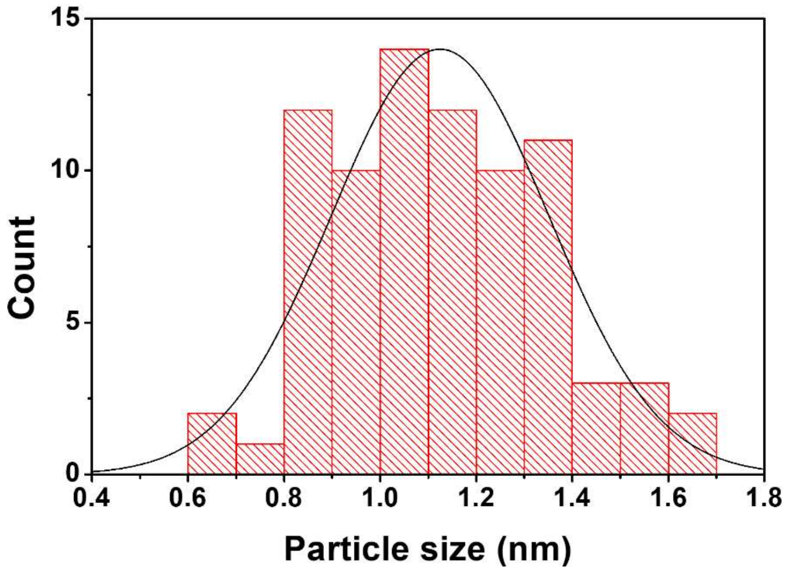

Figure S21. Size distribution of freshly prepared H-SiO_2@Ru@SiO_2-50.

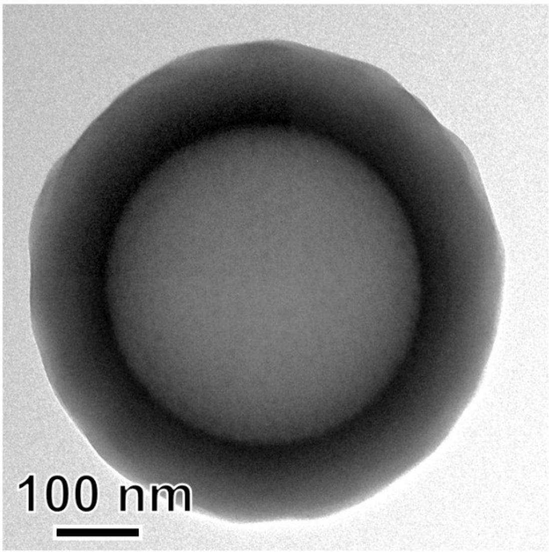

Figure S22. TEM image of reduced H-SiO_2@Ru@SiO_2-50, denoted as H-SiO_2@Ru@SiO_2-50-H_2.

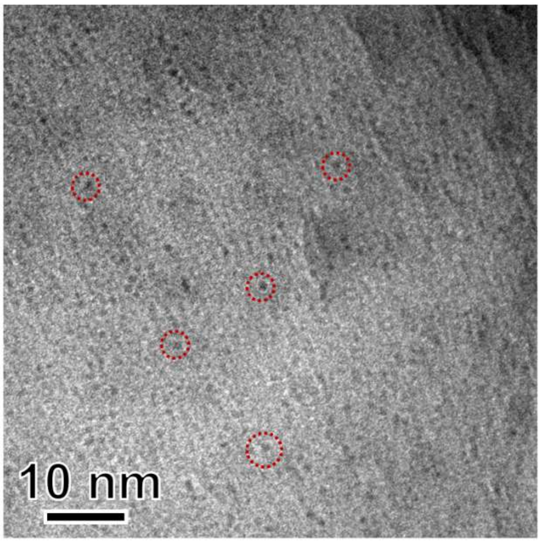

Figure S23. High-resolution TEM image of H-SiO_2@Ru@SiO_2-50-H_2.

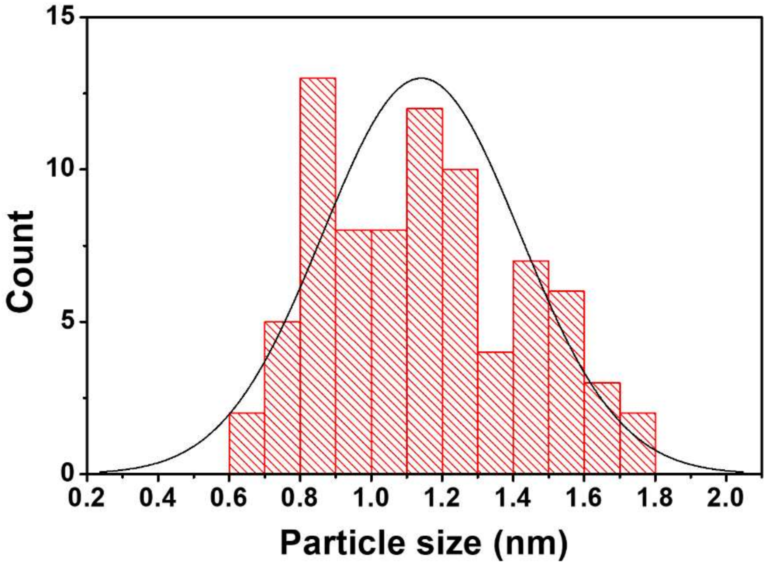

Figure S24. Size distribution of H-SiO_2@Ru@SiO_2-50-H_2.

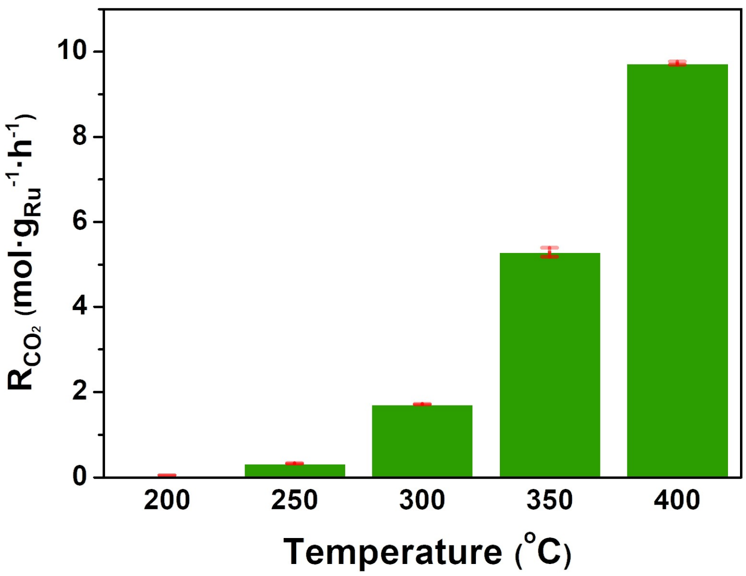

Figure S25. Temperature dependent activity of H-SiO_2@Ru@SiO_2-50-H_2 in catalyzing CO_2 hydrogenation.

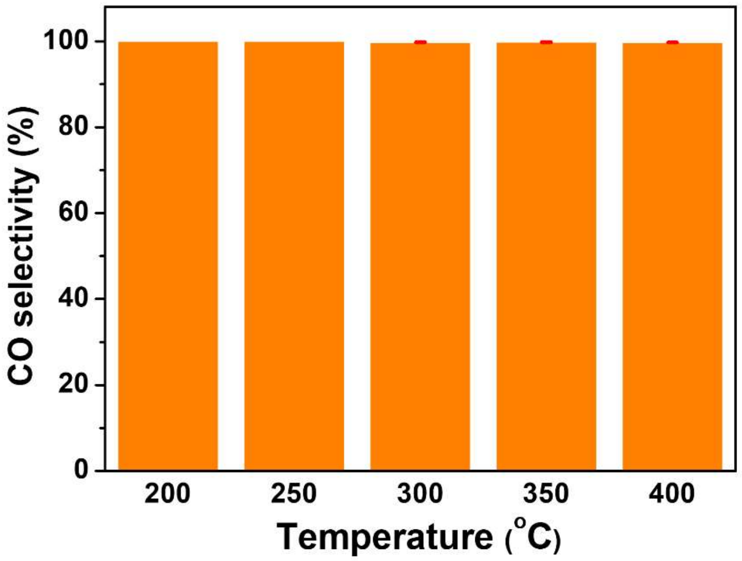

Figure S26. Temperature dependent selectivity of H-SiO_2@Ru@SiO_2-50-H_2 in catalyzing CO_2 hydrogenation.

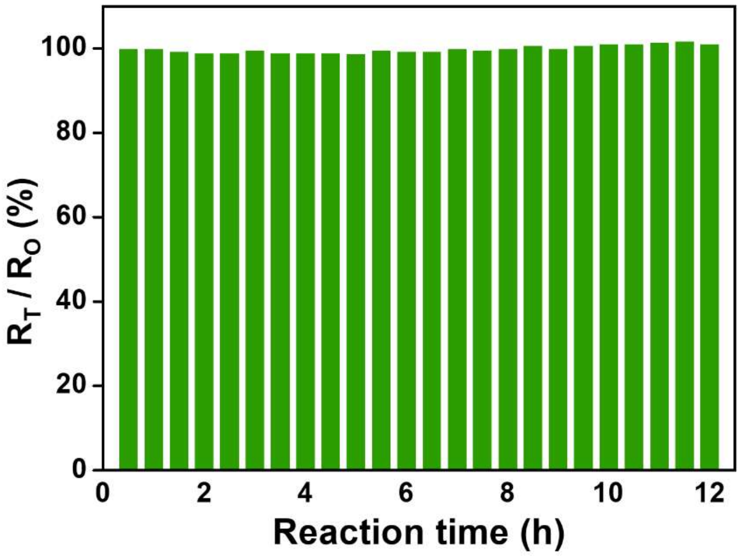

Figure S27. Catalytic stability of H-SiO_2@Ru@SiO_2-50-H_2 in a continuous 12-hour run at 400 °C.

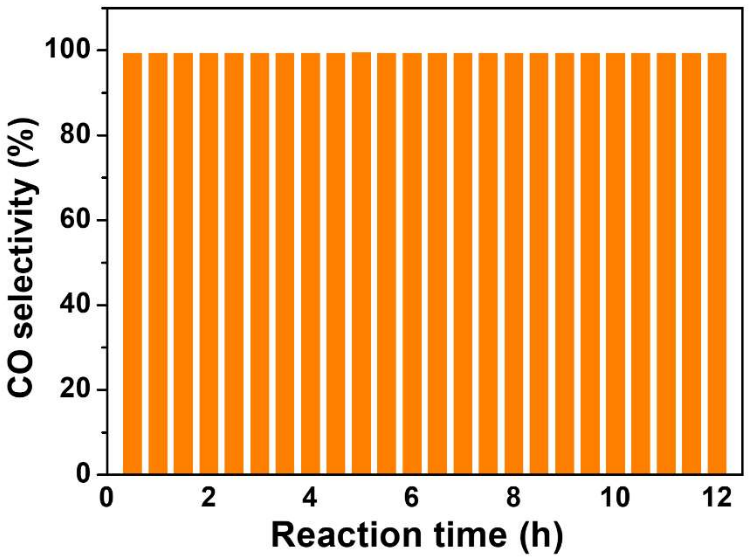

Figure S28. Catalytic selectivity of H-SiO_2@Ru@SiO_2-50-H_2 in a continuous 12-hour run at 400 °C.

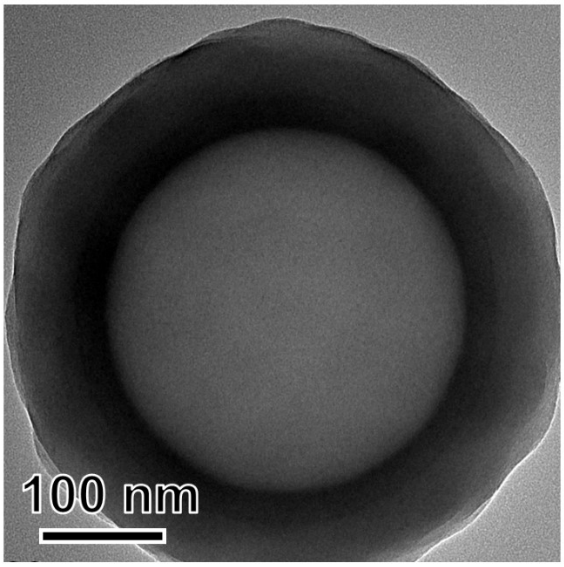

Figure S29. TEM images of spent H-SiO_2@Ru@SiO_2-50-H_2 after test at 400 °C for 12h.

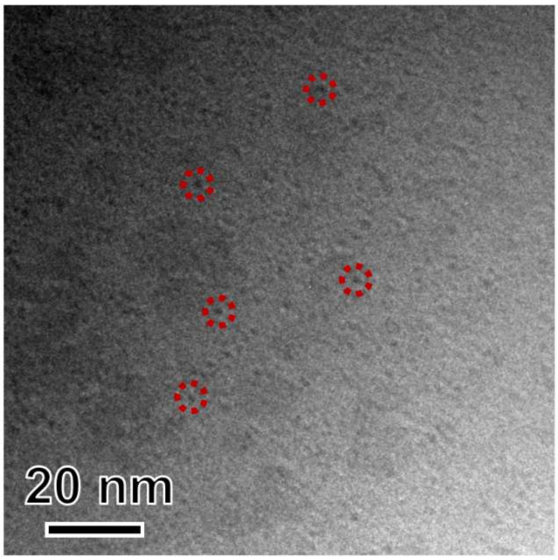

Figure S30. High-resolution TEM images of spent H-SiO_2@Ru@SiO_2-50-H_2 after test at 400 °C for 12h.

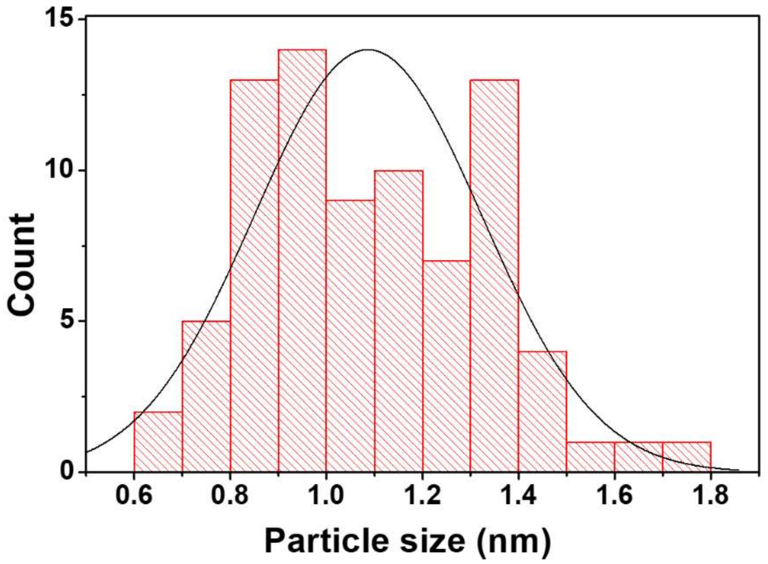

Figure S31. Size distribution of spent H-SiO_2@Ru@SiO_2-50-H_2.

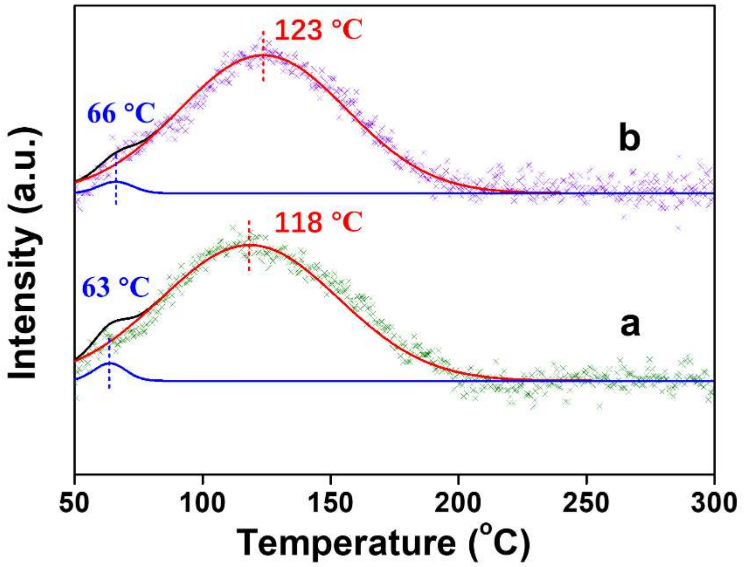

Figure S32. Hydrogen temperature programmed desorption (H_2-TPD) profile: (a) H-SiO_2@Ru@SiO_2-50-H_2, (b) spent H-SiO_2@Ru@SiO_2-50-H_2.

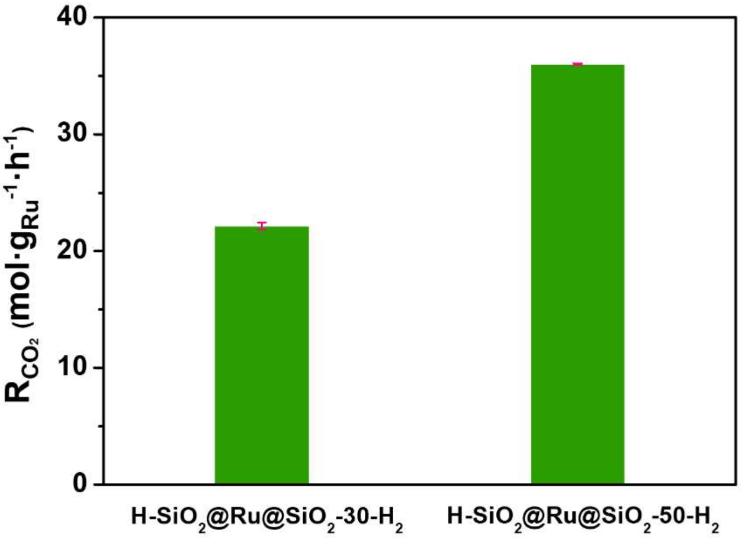

Figure S33. Catalytic activity comparison between H-SiO_2@Ru@SiO_2-30-H_2 and H-SiO_2@Ru@SiO_2-50-H_2 at 500 °C.

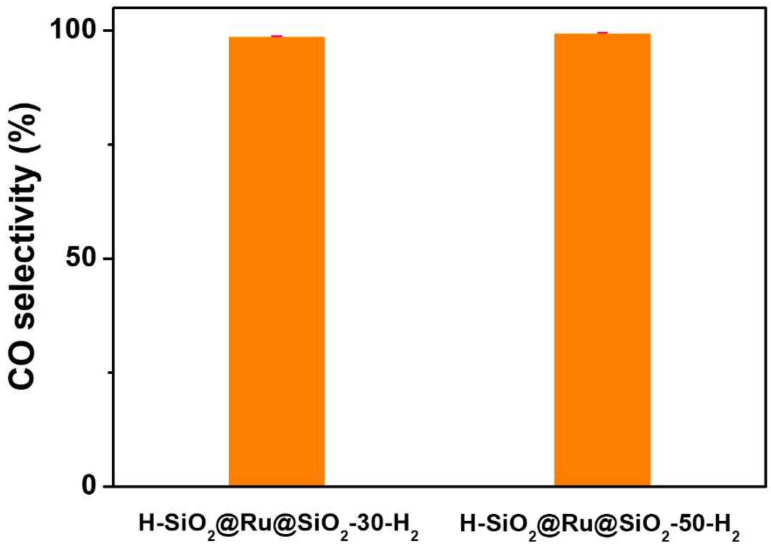

Figure S34. Catalytic selectivity comparison between H-SiO_2@Ru@SiO_2-30-H_2 and H-SiO_2@Ru@SiO_2-50-H_2 at 500 °C.

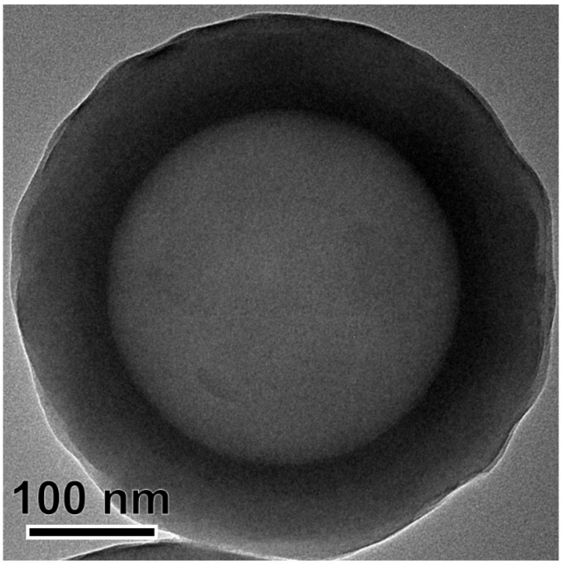

Figure S35. TEM images of H-SiO_2@Ru@SiO_2-50-H_2 after pretreated at 800 °C in H_2, denoted as H-SiO_2@Ru@SiO_2-50-800.

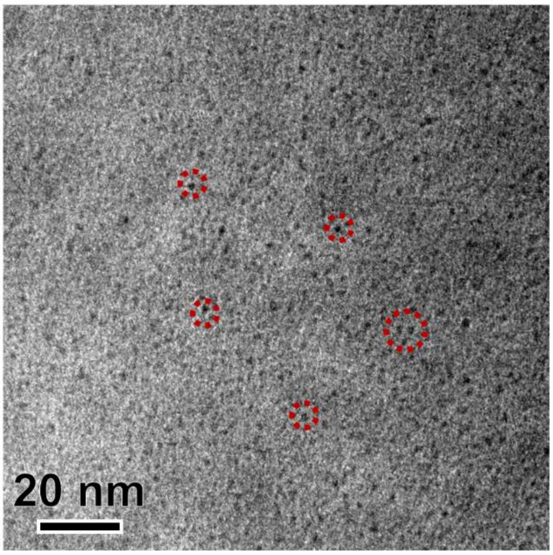

Figure S36. High-resolution TEM images of H-SiO_2@Ru@SiO_2-50-800.

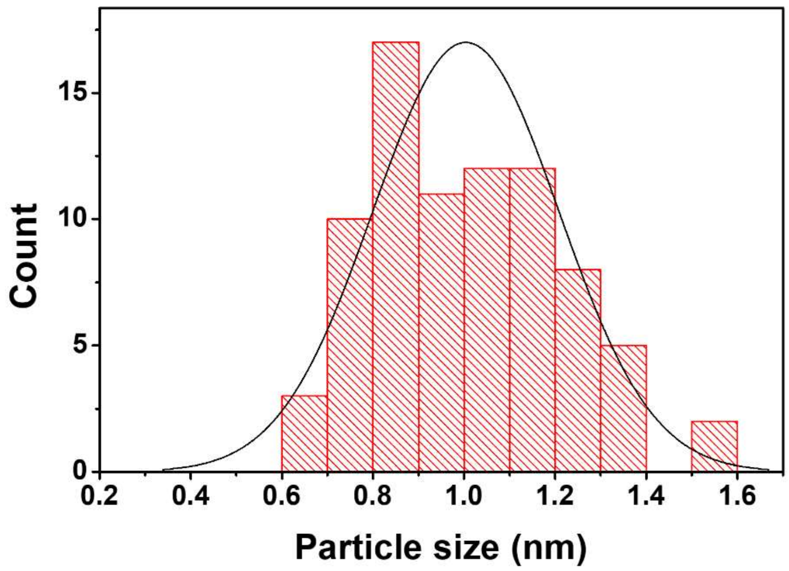

Figure S37. Size distribution of H-SiO_2@Ru@SiO_2-50-800.

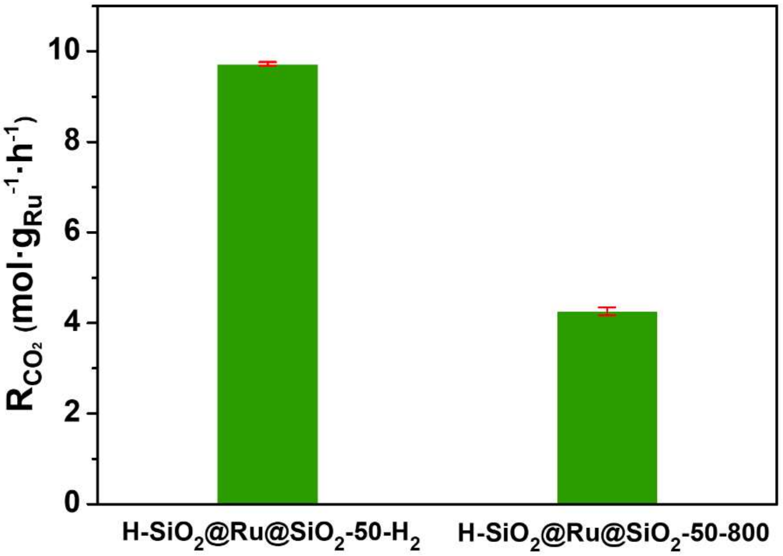

Figure S38. Catalytic activity comparison between H-SiO_2@Ru@SiO_2-50-H_2 and H-SiO_2@Ru@SiO_2-50-800.

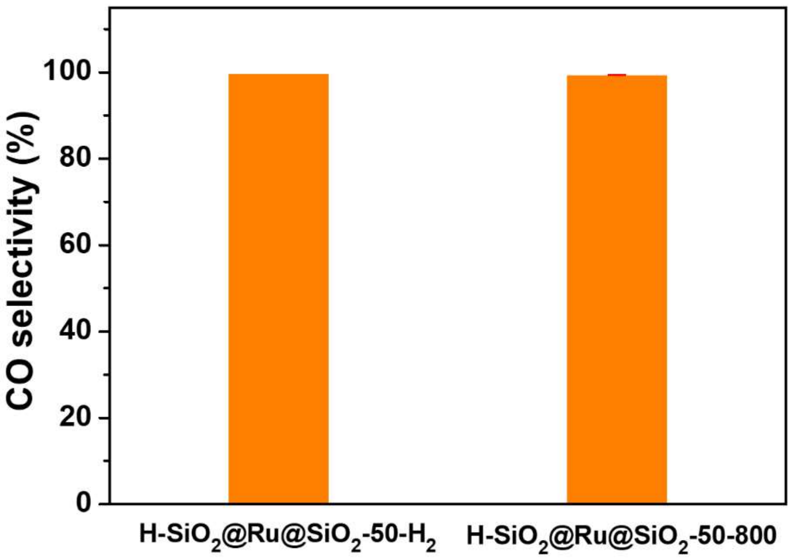

Figure S39. Catalytic selectivity comparison between H-SiO_2@Ru@SiO_2-50-H_2 and H-SiO_2@Ru@SiO_2-50-800.

Table S1. Comparison of Ru-based catalysts for hydrogenation of CO_2.

catalyst	Loading[a] (%)	Temp (°C)	CO sel. (%)	TOF(s^{-1})	Ref.
0.4%Ru@mSiO_2-N	0.4	400	93.4	0.127[c]	1
1.6%Ru@mSiO_2-N	1.6	400	83.5	0.053[c]	1
2.7%Ru@mSiO_2-N	2.7	400	72.4	0.035[c]	1
1.4%Ru/TiO_2	1.4	200	0	0.025[c]	2
2.0%Ru/SiO_2	2	350	47	0.054[c]	3
0.1%Ru/Al_2O_3	0.1	350	34.8	0.112[c]	4
$(Co_{0.95}Ru_{0.05})_3O_4$	5.0	300	1	-	5
$Ce_{0.99}Ru_{0.01}O_2$	1.0	500	10	0.12[c]	6
Ru/FeO_x	0.01	250	100	0.043[b]	7
Ru/a-TiO_2	1	300	99	-	8
Ru/MnO_x	5	300	10	0.5[b]	9
Ru/CeO_2	0.5	260	90	0.0055[b]	10
H-SiO_2@Ru@SiO_2-30	0.48	350	99.4	0.109[b]	This work
H-SiO_2@Ru@SiO_2-30	0.48	400	99.3	0.262[b]	This work

[a] Ru loading was measured by ICP-MS.

[b] TOF was calculated as number of CO_2 converted per Ru active site per second.

[c] TOF was calculated as mole of CO_2 converted per mole of total metal per second.

Reference

(1) Dou, J.; Sheng, Y.; Choong, C.; Chen, L.;Zeng, H. C. Silica nanowires encapsulated Ru nanoparticles as stable nanocatalysts for selective hydrogenation of CO_2 to CO. *Appl. Catal. B* **2017,** *219*, 580-591.

(2) Li, C.; Zhang, S.; Zhang, B.; Su, D.; He, S.; Zhao, Y.; Liu, J.; Wang, F.; Wei, M.; Evans, D. G., et al. Photohole-oxidation-assisted anchoring of ultra-small Ru clusters onto TiO_2 with excellent catalytic activity and stability. *J. Mater. Chem. A* **2013,** *1*, 2461-2467.

(3) Scire, S.; Crisafulli, C.; Maggiore, R.; Minico, S.;Galvagno, S. Influence of the support on CO_2 methanation over Ru catalysts: an FT-IR study. *Catal. Lett.* **1998,** *51*, 41-45.

(4) Kwak, J. H.; Kovarik, L.;Szanyi, J. CO_2 Reduction on Supported Ru/Al_2O_3 Catalysts: Cluster Size Dependence of Product Selectivity. *ACS Catal.* **2013,** *3*, 2449-2455.

(5) Zhu, Y.; Zhang, S.; Ye, Y.; Zhang, X.; Wang, L.; Zhu, W.; Cheng, F.;Tao, F. Catalytic Conversion of Carbon Dioxide to Methane on Ruthenium–Cobalt Bimetallic Nanocatalysts and Correlation between Surface Chemistry of Catalysts under Reaction Conditions and Catalytic Performances. *ACS Catal.* **2012,** *2*, 2403-2408.

(6) Sharma, S.; Hu, Z.; Zhang, P.; McFarland, E. W.;Metiu, H. CO_2 methanation on Ru-doped ceria. *J. Catal.* **2011,** *278*, 297-309.

(7) Zhang, D.; Luo, J.; Wang, J.; Xiao, X.; Liu, Y.; Qi, W.; Su, D. S.;Chu, W. Ru/FeOx catalyst performance design: Highly dispersed Ru species for selective carbon dioxide hydrogenation. *Chin. J. Catal.* **2018,** *39*, 157-166.

(8) Li, X.; Lin, J.; Li, L.; Huang, Y.; Pan, X.; Collins, S. E.; Ren, Y.; Su, Y.; Kang, L.; Liu, X., et al. Controlling CO_2 Hydrogenation Selectivity by Metal-Supported Electron Transfer. *Angew. Chem. Int. Ed.* **2020,** *59*, 19983-19989.

(9) Dreyer, J. A. H.; Li, P.; Zhang, L.; Beh, G. K.; Zhang, R.; Sit, P. H. L.;Teoh, W. Y. Influence

of the oxide support reducibility on the CO_2 methanation over Ru-based catalysts. *Appl. Catal. B* **2017,** *219*, 715-726.

(10) Aitbekova, A.; Wu, L.; Wrasman, C. J.; Boubnov, A.; Hoffman, A. S.; Goodman, E. D.; Bare, S. R.;Cargnello, M. Low-Temperature Restructuring of CeO_2-Supported Ru Nanoparticles Determines Selectivity in CO_2 Catalytic Reduction. *J. Am. Chem. Soc.* **2018,** *140*, 13736-13745.

Table S2. Properties and catalytic performance of different catalysts

Catalyst	Shell thickness (nm)	Ru loading (%)	Temperature (°C)	CO selectivity (%)	Conversion rate (mol/g/h)	Conversion degree (%)
H-SiO_2@Ru	0	0.99	200	83.9	0.04	3.2%
			250	83.6	0.26	3.3%
			300	85.4	1.46	3.9%
			350	83.8	4.50	6.0%
			400	83.1	10.15	9.5%
H-SiO_2@Ru@SiO_2-30	30	0.48	200	100	0.05	<1%
			250	99.5	0.19	<1%
			300	99.4	0.86	1.2%
			350	99.4	3.08	1.9%
			400	99.3	7.40	3.7%
H-SiO_2@Ru@SiO_2-50	50	0.27	200	100	0.05	2.2%
			250	100	0.32	2.7%
			300	99.7	1.72	3.2%
			350	99.8	5.28	3.9%
			400	99.7	9.72	4.6%
H-SiO_2@Ru@SiO_2-50-800	50	0.27	400	99.4	4.26	2.2%

Table S3. Ru dispersity of different catalysts

Catalyst	H_2 uptake[a]	Ru dispersion[b]	Size Range of Ru[c]
H-SiO_2@Ru-H_2	28.3	58%	1.0~6.0 nm
spent H-SiO_2@Ru-H_2	9.4	17%	1.0~12.0 nm
H-SiO_2@Ru@SiO_2-30-H_2	18.7	79%	0.7~1.8 nm
spent H-SiO_2@Ru@SiO_2-30-H_2	17.7	75%	0.6~1.7 nm
H-SiO_2@Ru@SiO_2-50-H_2	11.4	85%	0.6~1.7 nm
spent H-SiO_2@Ru@SiO_2-50-H_2	10.3	77%	0.6~1.8 nm

[a]Unit: μmoles H_2/g catalyst.

[b]Calculated based on the adsorption of one H atom per exposed Ru atom.

[c]Particle sizes obtained from TEM images.
